# A multimodal biomechanics dataset with synchronized kinematics and internal tissue motions during reaching

**DOI:** 10.1038/s41597-026-07019-3

**Published:** 2026-03-18

**Authors:** Roger Pallarès-López, Duarte Folgado, Uriel Magana-Salgado, Jessica Rosendorf, Enya Ryu, Micha Feigin-Almon, Hugo Gamboa, Luca Daniel, Brian W. Anthony, Praneeth Namburi

**Affiliations:** 1 Department of Mechanical Engineering, MIT, Cambridge, MA 02139 USA; 2 Fraunhofer Portugal AICOS, Porto, 4200-135 Portugal; 3 Comprehensive Health Research Center (CHRC), Porto, 4200-135 Portugal; 4 Department of Electrical Engineering and Computer Science, MIT, Cambridge, MA 02139 USA; 5 Research Laboratory for Electronics, MIT, Cambridge, MA 02139 USA; 6 Institute for Medical Engineering and Science, MIT, Cambridge, MA 02139 USA; 7 MIT.nano Immersion Lab, MIT, Cambridge, MA 02139 USA

## Abstract

Tissue motions within body segments, such as the relative movements of muscles, fascia, and bone, remain largely unexplored despite their relevance to movement dysfunction, force transmission, and motor skill. Here, we present a time-synchronized multimodal dataset that bridges this gap by capturing both internal tissue dynamics and conventional biomechanical measurements during arm reaching. Thirty-six participants across three expertise levels (world-class athletes, regional athletes, and untrained individuals) performed slow, rhythmic reaching movements while we recorded data using B-mode ultrasound imaging, motion capture, electromyography, and accelerometry. The dataset includes processed signals, derived parameters (segmented reach events, tissue boundary motion, arm kinematics, tremor events, and muscle activation levels), and metadata. Notably, using the DUSTrack point-tracking workflow, we provide trajectories for 11 points across approximately 300,000 ultrasound frames from the upper arm. This resource enables at least three primary applications: (1) supervised training and benchmarking of deep learning models for point tracking in ultrasound videos, (2) development of ultrasound-based metrics for characterizing soft tissue mechanics, and (3) biomechanical investigation of how tissue-level dynamics support motor performance. All data, processing code, and tutorials are provided in accessible formats with documentation.

## Background & Summary

Human movement is the functional output of dynamic interactions between the brain’s neural commands and the body’s musculoskeletal system. External kinematic measurements, such as joint angles, are commonly used to quantify the motions of body segments. However, these external measurements treat body segments as rigid units, thereby overlooking the complex, independent sliding and deformation of internal structures. Within each body segment, tissues such as muscles, fascia, and tendons, slide and move relative to one another. These ***differential tissue motions*** are not captured directly by external kinematics, and understanding them could inform mechanisms underlying movement dysfunction^1–3^, force transmission in the body^4–6^, and general motor skill^7^. Yet, compared to kinematics and neuromuscular data^8–17^, there is a paucity of data on differential tissue motions within body segments (Supplementary Table 1).

Reaching has served as a model behavior for studying planning, adaptation, and coordination in sensorimotor control^18,19^. Experimental designs use various constraints, such as restricting movement to planar (2D) setups with robotic exoskeletons, bracing joints to limit shoulder/wrist mobility, or requiring movements to predefined static or moving targets^20–22^. These approaches reveal different aspects of feedforward planning, online feedback, and motor learning. While prior work has characterized segment-level kinematics alongside neural population activity and electromyography (EMG) patterns during reaching^23–26^, the differential tissue motions within a limb segment remain largely unexplored^27^ (Supplementary Table 2).

Here, we address this gap and connect internal tissue dynamics to conventional biomechanical measurements (body segment kinematics and EMG) by introducing a multimodal time-synchronized dataset on reaching. This dataset includes ultrasound videos, motion capture, EMG, and accelerometry collected during an unconstrained arm reaching task. This dataset was obtained from 36 participants across three expertise levels: world-class experts, regional-level athletes, and untrained non-experts. We obtained arm kinematics using optical motion capture and observe internal tissue motions through ultrasound imaging and accelerometry. We also provide several measurements extracted from ultrasound videos using a point-tracking workflow^28^. These include movements of the brachialis-triceps muscle boundary, humerus bone motion, and movements within the triceps and brachialis muscles. From the ultrasound point trajectories, higher-order parameters can be derived, including tissue deformation and muscle area changes during reaching.

We adopt a point-tracking approach because of its versatility. Point trajectories can be used to derive higher-order parameters such as shear, divergence, and in-plane rotational components (torsion) that can further shed light on mechanisms by which muscles and associated soft tissues give rise to movement.

Specifically, the dataset includes synchronized: (1) B-mode ultrasound videos providing a transverse view of the triceps and brachialis muscles and surrounding tissues, (2) 11 frame-level tracked points in the ultrasound videos including two points on the humerus bone, points within the triceps and brachialis muscles, and points at the boundary between these muscles, (3) 3D optical motion capture from five upper-limb landmarks, (4) tri-axial accelerometer and EMG data from the palm, biceps, and triceps, (5) start/end times of trials, reach cycles, and tremor events, and (6) participant metadata, including demographics, expertise level and experimental configuration.

In this paper, we present a thorough validation of our synchronization protocols, detailed descriptions of ultrasound probe placements, validation metrics, and typically computed parameters. We also provide algorithms to transform source data into these derived measurements. The data is provided in commonly used open-source formats, with tutorials in Python and MATLAB. These accompanying tutorials show how to load the files, navigate the dataset structure, and visualize synchronized time series data across modalities alongside the event annotations. They also demonstrate how to overlay tracked points on ultrasound video frames and export annotated videos, providing a practical starting point for downstream biomechanical analyses and machine-learning workflows.

### Example applications

This dataset supports several applications in biomechanics, muscle modeling, signal processing, and deep learning. These include relating differential tissue motions with expertise, estimating muscle motion in three dimensions, quantifying differential motions between tissue layers within the imaging plane, development of ultrasound-based metrics for characterizing soft tissue motions, development of signal processing algorithms related to tremor detection, and developing and benchmarking deep learning models for ultrasound point tracking.

The signal processing application (tremor detection) is demonstrated in this manuscript. Related publications^7,28^ have demonstrated all other uses (see Related publications), confirming the dataset’s practical utility across multiple research contexts. However, these reports represent initial explorations, leaving considerable scope for further investigation—with the deep learning application offering a particularly novel direction for utilizing this dataset.

This multimodal dataset supports the investigation of differential tissue motions in relation to external kinematics, neuromuscular activity, and inter-individual variability across skill levels^7^. Therefore, researchers in motor control and biomechanics can use the dataset to investigate how internal tissue dynamics relate to joint-level kinematics and movement expertise.

Biomechanical models often treat muscles as massless actuators to simplify analysis^29^. However, muscles have intrinsic 3D structure and exhibit inertial effects along with heterogeneous, anisotropic elasticity^30^. To understand these features, it is essential to quantify muscle motions. This dataset can be used to jointly examine inferred muscle motion along the line of actuation, muscle motion within the imaging plane orthogonal to actuation, and subtle movement-induced tremors. It offers a more complete view of muscle dynamics than one-dimensional measurements like fascicle length estimates, and initial insights demonstrating this application are reported elsewhere^7^.

In addition, point tracking in ultrasound videos can be used to quantify differential motions between layers of the body, including the skin, superficial fascia^31,32^, deep fascia^33^, the epimysium of the muscles, and deep connective tissues such as the interosseous membrane^34^. Within each layer, these measures could support quantification of boundary kinematics (e.g., between muscles), as well as elongation and shortening, to determine how different layers of the body interact with one another and contribute to movement.

To our knowledge, this is the first dataset to provide continuous frame-level tracking for points in ultrasound videos. Existing ultrasound datasets with tracked anatomical features (e.g. fascicle length) typically provide manual annotations at sparse frames^35,36^, rather than offering frame by frame annotations, mostly due to the time-consuming and expertise-dependent nature of identifying correspondence across frames in ultrasound data. This limitation and challenge are well-recognized in the field of ultrasound tracking^37,38^. Our dataset provides trajectories for 11 tracked points across almost 300,000 frames. The medical imaging and machine learning communities can use the tracked point trajectories for supervised training and benchmarking of deep learning models—from muscle-specific pipelines to general-purpose track-any-point (TAP) models adapted from video tracking—with the goal of achieving automated point tracking in ultrasound^35,37–39^.

This dataset is therefore a valuable resource for advancing both the understanding of muscle function and the development of deep-learning-based tracking methods for medical ultrasound imaging.

### Related publications

Portions of this dataset^40^ have been used in previous publications. Specifically, our companion study “Efficient elastic tissue motions indicate general motor skill” reports analyses of expertise-related differences in reaching^7^. Additionally, “DUSTrack: Semi-automated point tracking in ultrasound videos” utilizes the ultrasound videos and tracked point trajectories from this dataset to describe and evaluate the point-tracking workflow^28^.

## Methods

### Participants

Data were collected from 36 healthy adult participants (age: 26.1 ± 7.3 years; 15 identified as male, 19 as female, and 2 as non-binary). Participants were recruited based on their prior experience with movement disciplines and were categorized into three expertise groups: 11 experts, 14 intermediates, and 11 non-experts (Table 1). Participants had to be healthy, with no known underlying cardiac or respiratory conditions. Additionally, candidates for the intermediate and expert groups with a current or recent injury that prevented them from practicing their discipline were excluded.

**Table 1:** Participant demographics and anthropometrics summary.

Fields	Statistic
Participant Number	36
Age (yr)	26.1 ± 7.3
Gender	41.7% M, 52.8% F, 5.5% O
Height (cm)	171.6 ± 9.0
Weight (kg)	65.4 ± 10.8
Arm Length (cm)	66.0 ± 4.6
Muscle Thickness (mm)	13.4 ± 4.6
Expertise Level	30.6% E, 38.8% I, 30.6% N
Dominant Side	88.9% R, 11.1% L
Probe Side	47.2% R, 52.8% L

Summary statistics for the 36 participants included in the dataset. Numerical variables are reported as mean ± standard deviation, and categorical variables are reported as percentages. Abbreviations: M = male, F = female, O = other; E = expert, I = intermediate, N = non-expert; R = right, L = left.

Expertise level was based on their training history and recognized achievements in sports or performance disciplines. Individuals were classified as experts if they had attained national or international-level recognition in their field, such as competing in the Olympics, national championships (e.g., NCAA), or performing on global stages (e.g., Broadway productions). All experts were actively practicing their discipline at the time of data collection. The expert group included participants from diverse domains such as professional dance—Ballroom (2), Ballet (1), Bharatanatyam (1), Latin (1), Hip-hop (1), Modern (1); individual and team sports—Squash (1), Volleyball (1), Rowing (1); and theatrical performance—Broadway (1). Intermediates were individuals with some formal training, such as regional-level athletes or long-term recreational practitioners, who did not meet the criteria for expert classification. Non-experts were participants with no formal training or systematic engagement in any movement-related discipline. Competitive accomplishments were self-reported, and were not independently verified via external records (e.g., certifications or competition databases).

### Experimental protocol

The experimental task involved unconstrained, goal-agnostic reaching movements that could be performed similarly across participants with varying levels of expertise. The overarching design objective was to minimize the influence of factors that are often shaped by training in specific disciplines (such as task-specific strategies, reaction times, or anticipatory movements^41^) and instead reveal more general, internal features of movement execution.

Participants performed slow, rhythmic forward-reaching arm movements while standing upright. Each movement began with the hand positioned next to the hip, extended forward in the sagittal plane, and then returned to the starting position. This forward and backward motion constituted one full reach cycle. To explore natural variations in hand orientation, participants completed the task under two conditions: “give” (palm facing up) and “touch” (palm facing down). Participants were instructed to move as if handing over an object in the “give” condition or touching an object in front of them in the “touch” condition. Participants were instructed to start and end each reach cycle with their hand next to their hip and reach forward. They were also told not to pause at the transitions between extension and retraction. No additional constraints were imposed on the joint trajectory. Participants practiced the reaching motion 1-2 times and were reminded of the instructions if they deviated during practice. Each participant then performed the task—approximately 10 repetitions of both the give and touch conditions. The experimenter verbally indicated when to switch between the “give” (palm up) and “touch” (palm down) conditions. No feedback or correction was provided during the task to preserve natural variation in movement execution.

To standardize movement tempo and reduce variability in kinematic execution across participants, a visual metronome was displayed on a monitor in front of the participant. The metronome consisted of a dot oscillating vertically at 1/6 Hz (1 cycle every 6 seconds), guiding the extension and retraction phases with equal timing (3 seconds each). Participants were asked to synchronize their arm movements with this pacing, initiating extension when the dot rose and retraction as it fell. No auditory cues or haptic feedback were used.

The choice of a slow pacing frequency was intentional. Prior literature suggests that humans exhibit reduced motor precision and increased difficulty when performing very slow movements, especially in the absence of clear goals or feedback^42,43^. By imposing a slow, goal-free movement context, the task was designed to expose subtle differences in internal muscle control and dynamics that may not be evident under typical movement speeds or goal-directed conditions. These differences are reported elsewhere^7^.

### Experimental procedures

On the day of the experiment, participants arrived at the laboratory and underwent consenting procedures. Participants were introduced to the study goals, informed about the experimental procedures, and given an opportunity to ask questions. They then completed a questionnaire on their movement background, which was used to classify them into one of the three expertise categories (expert, intermediate, non-expert), as detailed in the Participants section.

Then, participants were guided through the sensor placement process. Five reflective motion capture markers were affixed to anatomical landmarks on the upper limb using kinesiology tape (Hampton Adams): shoulder (acromion), upper arm (deltoid insertion into the humerus), elbow (lateral epicondyle), forearm (midpoint between the distal end of the ulna and the lateral epicondyle of the elbow), and hand (distal portion of the second or third metacarpal on the dorsal surface). After the motion markers were secured, three Delsys Trigno wireless sensors were placed on the biceps, triceps, and abductor pollicis brevis (palm) to capture surface EMG and tri-axial accelerometer data.

Finally, a linear ultrasound probe was positioned on the participant’s upper arm to capture transverse cross-sectional views of the triceps and brachialis muscles. The probe was secured using a custom 3D-printed mount and self-adhesive wrap, with standard ultrasound gel applied between the probe and skin to ensure acoustic coupling. Probe/gel temperature was not controlled, and pressure under the probe was not measured. However, participants were asked to provide feedback on the comfort and tightness of the probe attachment, and adjustments were made as needed. Experimenters ensured that the probe was neither too loose (to avoid slippage) nor too tight (to avoid restricting movement or circulation).

Before data collection began, participants were given an opportunity to get accustomed to moving with the sensors on their body by practicing the reaching task a few times. This allowed them to become familiar with the required movement while wearing the full sensor setup. The arm used for reaching was randomly assigned to avoid systematic effects of limb dominance. Assignment was block-randomized in groups of 20 participants to promote balance between right and left probe-side conditions.

### Data collection

**Fig. 1:**
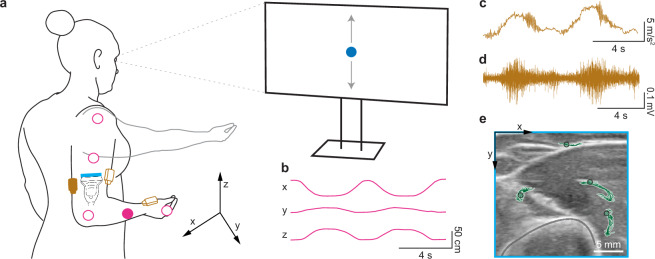
Experimental setup and example multimodal recordings. (**a**) Schematic of the experimental setup showing a participant performing the arm-reaching task while wearing reflective infrared reflective markers (pink), wireless Delsys EMG/IMU sensors (light brown), and an ultrasound probe placed transversely on the upper arm (light blue), while following the visual metronome (blue dot on the screen). The laboratory coordinate frame is shown for reference, with the z-axis pointing upward. Data from solid colored sensors are shown in panel b (pink) and panels c, d (brown). (**b**) Example trajectory of the forearm marker captured via motion capture, with x, y, and z coordinates shown over time. (**c**) Example x-axis acceleration signal from a Delsys Avanti sensor on the triceps, showing visible tremor oscillations. (**d**) Example raw EMG trace from the same triceps sensor. (**e**) Representative B-mode ultrasound frame recorded with the Cephasonics system, overlaid with tracked point paths of four points to illustrate tissue motion across frames. The pixel coordinate frame is shown for reference.

Multimodal data were collected using three primary sensing systems: optical motion capture, ultrasound imaging, and wireless EMG with accelerometry (Fig. 1). Synchronization signals were used to align data across modalities (see Data Synchronization).

Upper-limb kinematics were recorded using an optical motion capture system (OptiTrack NaturalPoint, Inc., Corvallis, OR, USA) using 28 Prime 13 cameras operating at 240 Hz (Fig. 1a,b). Four cameras captured reference videos for data cleaning and annotation, while 24 cameras were used for tracking. Five infrared reflective markers were placed on the key anatomical landmarks described in Experimental Procedures (shoulder, elbow, forearm, wrist and hand). Marker positions were recorded using Motive software (versions 2.2.0, 3.0.1, and 3.0.2) and exported in CSV (Comma-Separated Values) format.

Internal tissue motions were captured using an ultrasound imaging system (Fig. 1a,e): a Cephasonics Cicada system (Cephasonics Ultrasound Solutions, San Jose, CA) equipped with a 7.5 MHz, 128-element linear array transducer. The probe, 3.75 cm in width, was positioned transversely on the upper arm to acquire cross-sectional B-mode images of the triceps and brachialis muscles. The depth was set to 5 cm and the focal depth to 3 cm. The acquired image resolution was approximately 75.6 µm/pixel (~13 pixels per mm). Ultrasound frames were recorded at a native acquisition rate of approximately 62 frames per second. Each session produced frame-indexed PNG image sequences, accompanied by UNIX timestamp metadata.

Cephasonics imaging parameters were held constant across participants using a fixed acquisition setup (i.e., gain-related and display settings were not adjusted on a per-participant basis). The configuration file used for acquisition is provided with the dataset for reference and reproducibility.

Surface electromyography and inertial data were collected using Delsys Trigno wireless sensors (Delsys Inc., Natick, MA) (Fig. 1a,c,d). Sensors were placed on the triceps, biceps, and palm. Each sensor recorded both EMG and tri-axial accelerometer data. The accelerometer data were sampled at 148 Hz and the range set to ± 4 g, while EMG signals were sampled at 1259 Hz with a value range of 11 mV. Data acquisition and real-time signal visualization were managed using the Delsys Trigno Discover software (versions 1.4.2, 1.5.0, and 1.6.2) and exported in CSV format.

The Delsys system receives an integer number of samples every 13.5 ms. This should be considered when calculating the exact sampling rate of recorded signals. For example, if the reported sampling rate is 1259 Hz, the exact sampling rate would be round(1259 × 0.0135)/0.0135 ≈ 1259.259 Hz.

### Data synchronization

All sensing modalities (ultrasound, optical motion capture, and surface EMG/accelerometry) have their own clocks that were synchronized using hardware-based triggers and signal-level adjustments to ensure precise temporal alignment across systems.

The clock from the OptiTrack system was used as the reference clock for all data modalities. A *recording gate* Transistor-Transistor Logic (TTL) pulse was sent by the eSync2 timing module (NaturalPoint, Inc., Corvallis, OR, USA), with the high value indicating data capture state. The rising and falling edges of this TTL pulse started and stopped data recording in the Cephasonics ultrasound system, and this signal was sampled by the Delsys Trigno EMG system.

**Fig. 2:**
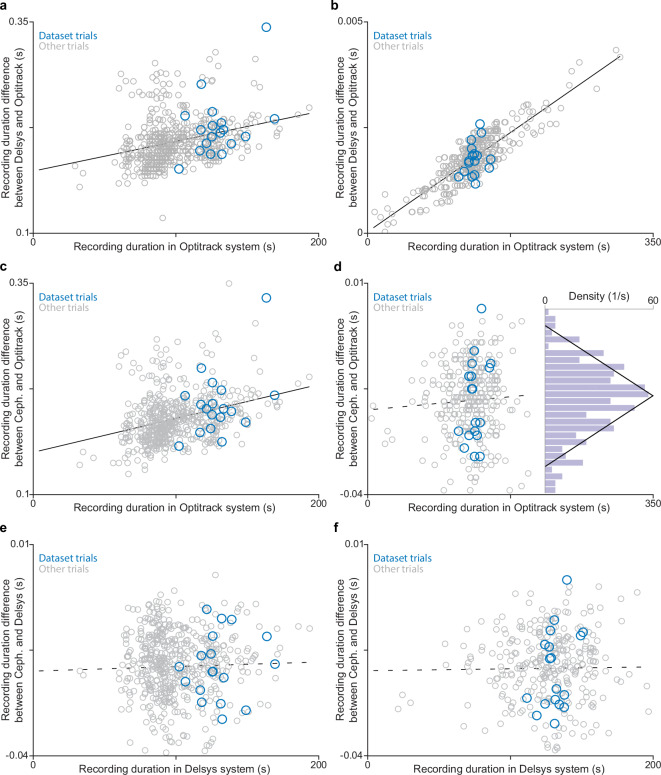
Time synchronization analysis used to assess clock drift and recording duration mismatches between OptiTrack and the other acquisition systems. (**a**–**f**) Comparison of recording durations across systems. Blue dots represent the trials in the presented dataset, gray dots represent trials captured with the same systems but for other studies. The solid and dashed black lines represent least-squares linear fits, with solid lines indicating statistically significant slopes and dashed lines indicating non-significant ones. (**a,b**) Comparison between OptiTrack duration (x-axis) and the difference between the TTL pulse duration on Delsys and the OptiTrack duration (y-axis). (**a**) Data acquired when using Motive 2.2.0. Linear regression fit parameters: slope = 0.0003523, CI = [0.0002583, 0.0004463], *p* = 6.83 × 10^−13^; intercept = 0.1734, CI = [0.1636, 0.1832], *p* = 3.35 × 10^-139^. (**b**) Data acquired when using Motive 3.0.1 and 3.0.2. Linear regression fit parameters: slope = 0.00001341, CI = [0.00001264, 0.000014196], *p* = 2.41 × 10^−115^; intercept = 0.00003189, CI = [−0.00007390, 0.0001377], *p* = 0.5537. (**c**,**d**) Comparison between OptiTrack duration (x-axis) and the difference between the Cephasonics video duration and the OptiTrack duration (y-axis). (**c**) Data acquired when using Motive 2.2.0. Linear regression fit parameters: slope = 0.0004003, CI = [0.0003052, 0.0004954], *p* = 1.07 × 10^−15^; intercept = 0.1496, CI = [0.1397, 0.1595], *p* = 1.52 × 10^−115^. (**d**) Data acquired when using Motive 3.0.1 and 3.0.2. Inset histogram depicting the duration difference following a triangular distribution (drawn in black for reference). Linear regression fit parameters: slope = 0.000017911, CI = [−0.00001549, 0.00005132], *p* = 0.29; intercept = −0.01998, CI = [−0.02432, −0.01565], p-value = 1.19 × 10^−17^. (**e,f**) Comparison between the TTL pulse duration on Delsys (x-axis) and the difference between Cephasonics video duration and the TTL pulse duration on Delsys (y-axis). (**e**) Data acquired when using Motive 2.2.0. Linear regression fit parameters: slope = 0.00001080, CI = [−0.00001942, 0.00004103], *p* = 0.48; intercept = −0.01996, CI = [−0.02312, −0.01680], *p* = 4.07 × 10^−31^. (**f**) Data acquired when using Motive 3.0.1 and 3.0.2. Linear regression fit parameters: slope = 0.00001072, CI = [−0.00002584, 0.00004729], *p* = 0.56; intercept = −0.02085, CI = [−0.02561, −0.01609], *p* = 3.08 × 10^−17^.

An analog input adapter (part DC-X06) in the Delsys Trigno system sampled the TTL pulse from eSync2 at 4444 Hz. This signal transitions to a high state when OptiTrack recording begins and returns to a low state when recording stops, marking the onset and offset of motion capture recording. Each hardware system has a different master clock, leading to a small but systematic difference in how the two systems measure time. For instance, relative to the Delsys clock, 240 Hz in OptiTrack may correspond to 239.997 Hz in Delsys. In this example, the ***effective clock multiplier*** between the two systems is 1.00001341. Consequently, the difference between the duration of the recorded TTL pulse in Delsys and the recording duration in OptiTrack scales linearly with recording duration. We performed a linear regression between these two parameters to determine the effective clock multiplier between OptiTrack, Delsys, and Cephasonics clocks (Fig. 2). We also included data from other experiments to improve the accuracy of this determination.

Based on personal communication with OptiTrack support, there are fundamental differences in how versions 2 and 3 of the Motive software handle file recording. This was reflected in our dataset, which is why we separately determined the clock multipliers for data acquired with different versions of Motive. For recordings acquired with Motive 2.2.0, the calculated clock multiplier was 1.0003523 (Fig. 2a). For Motive 3.0.1 and 3.0.2 recordings, the clock multiplier was 1.00001341 (Fig. 2b).

If there were no delays between triggering the TTL pulse and saving the recorded data in OptiTrack, we would expect the regression line to have a zero intercept. This is what we found for data collected using Motive 3 (Fig. 2b). Surprisingly, the intercept was 173.4 ms for data collected using Motive 2.2 (Fig. 2a). This suggests that the falling edge of the TTL pulse was delayed relative to the rising edge—meaning Motive stopped recording data before the falling edge of the TTL pulse was sent by eSync2. To verify this, we conducted an additional experiment using a Delsys Force-Sensitive Resistor (FSR) sensor that recorded the finger pressure profile as the researcher pressed a keyboard key to manually start and end each trial in OptiTrack. This confirmed that the duration difference originated from a delay in the TTL trigger’s falling edge (offset time), which caused both the Delsys and Cephasonics systems to terminate recording about 175 ms after the OptiTrack system. In contrast, both the rising and falling edges of the TTL pulse were well-aligned with the true start and end of the recording when using Motive 3.

Based on the estimated effective clock multipliers (Fig. 2, Table 2), we resampled and time-shifted all Delsys signals to align them with the OptiTrack timeline. We implemented this synchronization pipeline using custom Python scripts that used the pysampled package and performed the following steps: detected trigger events from the analog signal, applied temporal scaling based on the computed clock multiplier, shifted the Delsys data streams to align with OptiTrack based on the rising edge of the TTL pulse (with OptiTrack recording onset defined as 0 s), and saved the resampled Delsys data streams.

**Table 2:** Summary of cross-modal synchronization parameters and expected alignment uncertainty.

	Motive Version	OptiTrack (reference clock)	Delsys	Cephasonics
Clock Multiplier	2	1.0	1.0003523	1.0004003
	3	1.0	1.00001341	1.0
Offset Time (s)	2	0.0	0.1734	0.1496
	3	0.0	0.0	−0.01998
Uncertainty (ms)	2	0.0	26.5	27.6
	3	0.0	0.3	8.8

OptiTrack is treated as the master reference clock. For each Motive software version, the table reports the effective clock multiplier (relative clock scaling needed to map Delsys/Cephasonics time to the OptiTrack timeline), the estimated offset time relative to OptiTrack recording end, and an empirical uncertainty estimate based on the residuals of the duration-difference regression analyses.

The Cephasonics ultrasound system was placed in streaming mode before receiving the TTL pulse from OptiTrack’s eSync2, maintaining a buffer of B-mode ultrasound images in the computer’s RAM (Random Access Memory). An Arduino converted the rising and falling edges of the TTL pulse into simulated keypresses, marking the start and end frames in the buffer. Images from the start to end frame were written from RAM to the hard disk between each falling edge and the next rising edge of the TTL pulse (between trials).

We performed a similar synchronization analysis between the OptiTrack and Cephasonics systems (Fig. 2c,d). As with the OptiTrack-Delsys analysis, we found a duration difference between the OptiTrack and Cephasonics recordings. These values closely matched those from the OptiTrack-Delsys analysis, minus one Cephasonics frame (~17 ms). This one-frame difference occurs because Cephasonics is already streaming data when the rising edge of the TTL pulse from OptiTrack eSync2 arrives—which can happen at any point between two frame captures. The first frame will be marked *after* the TTL pulse arrives, creating an expected delay of half a frame. The same logic applies to the falling edge of the TTL pulse, adding another half-frame difference. Together, Cephasonics recordings are expected to be shorter than OptiTrack recordings by one Cephasonics frame on average. The mismatch is expected to follow a triangular distribution, spanning from 0 to 2 frames. This distribution originates from the sum of two uniformly distributed random variables (each between 0 and 1 frame) that represent the delays at the onset and offset of the TTL pulse (Fig. 2d, inset). Based on the Delsys-OptiTrack synchronization results (173.4 ms and 0 ms), the expected values when using Motive 2 and 3 are approximately 156 ms and −17 ms. Our analytical results (150 ms and −20 ms, Fig. 2c,d) align closely with these expectations.

The effective clock multiplier between the OptiTrack and Cephasonics systems was 1.0004003 when using Motive 2 and 1.0 when using Motive 3 (Fig. 2c,d). Since the point-tracking analysis was performed before computing these clock multipliers, we provide the corrected sampling rates for ultrasound videos and tracked point trajectories: 59.976 Hz for data collected using Motive 2 and 60 Hz for data collected using Motive 3. The time arrays included with the dataset account for this correction and should be used for cross-modal analyses.

Finally, we performed a similar duration-difference analysis between the Cephasonics and Delsys systems. Since both were driven by the same TTL pulse, we expect a duration difference of only one Cephasonics frame when using either Motive 2 or 3, based on the reasoning provided above. Our findings are consistent with this expectation (Fig. 2e,f).

The standard deviation of residuals from the linear regression in duration-difference analysis serves as an estimate of cross-modal alignment uncertainty. For data collected using Motive 2 and 3, these values are: 26.5 ms and 0.3 ms for analyses linking motion capture data with accelerometry/EMG data, 27.6 ms and 8.8 ms for analyses linking motion capture data with ultrasound data, and 8.4 ms and 9.2 ms for analyses linking accelerometry/EMG with ultrasound data. Consider these values when interpreting results from cross-modal analyses, such as cross-correlation. A summary of all relevant synchronization results is presented in Table 2.

### Data processing

Multimodal data from motion capture, surface EMG plus accelerometry, and ultrasound were processed using custom Python scripts and publicly available packages, including pysampled, numpy, and scipy. The following steps were applied to prepare the dataset for analysis and sharing.

**Fig. 3:**
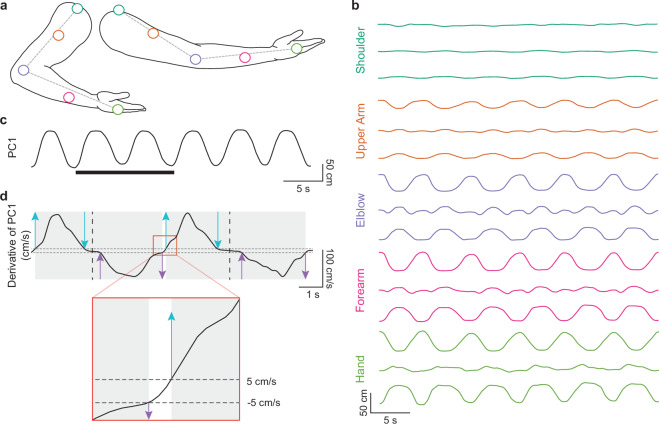
Reach-cycle segmentation from motion capture marker trajectories. Reach cycles were segmented using the time derivative of the first principal component (PC1) obtained from principal component analysis (PCA) of the five-marker kinematic data. (**a**) Locations of the five OptiTrack markers on the upper limb in representative retracted (left) and extended (right) postures. The dashed chain indicates the marker path used to estimate participant arm length (shoulder-elbow plus elbow-hand), which is provided for normalization. (**b**) Example 3D marker trajectories over six reach cycles. For each marker, traces show position along the anterior-posterior (top), medial-lateral (middle), and superior-inferior (bottom) axes. (**c**) At each time sample, the five 3D marker positions were concatenated into a 15-dimensional vector and PCA was applied. PC1 captures the primary reaching movement. The highlighted interval corresponds to the segment shown in panel d. (**d**) The time derivative of PC1 was used to identify extension and retraction phases. A ± 5 cm/s threshold (dashed lines) defined extension onset/offset and retraction onset/offset (arrows). Each reach cycle was defined from extension onset to retraction offset (gray boxes), and the extension-to-retraction transition time was defined as the midpoint between the threshold-based extension offset and retraction onset (dashed vertical line).

We cleaned optical motion capture data using Motive software. Gaps in marker trajectories caused by occlusion were corrected by merging discontinuous segments and interpolating missing data. Marker positions were exported in the OptiTrack global (laboratory) coordinate frame. During preprocessing, we converted the Motive-exported axes from the y-up convention to z-up by applying a fixed axis permutation (Fig. 1a). This operation is a rigid transformation that relabels axes without affecting inter-marker distances or kinematic features derived from the trajectories. The 3D positions of five tracked markers—shoulder, upper arm, elbow, forearm, and hand—were low-pass filtered at 10 Hz for further processing (Fig. 3).

To quantify arm motion, the 3D marker positions were concatenated into a 15-dimensional vector at each frame (Fig. 3b) and reduced to a 1D representation using principal component analysis (PCA). The first principal component (PC1) was sign-adjusted using the shoulder-to-hand vector so that extension corresponds to a positive slope (Fig. 3c). Arm velocity was computed as the first derivative of PC1 with a 5 Hz low-pass filter, and arm speed was defined as the absolute velocity (Fig. 3d).

Reach cycles were segmented by identifying threshold crossings in the arm velocity profile. A cycle began when arm velocity crossed +5 cm/s with positive slope (extension onset) and ended when velocity crossed –5 cm/s with positive slope (retraction completion). The extension-to-retraction transition was defined as the midpoint between negative-slope crossings of the ±5 cm/s thresholds (Fig. 3d). These events were detected automatically and manually verified through visual inspection to ensure consistency across trials.

Arm length for each participant was estimated using OptiTrack markers to normalize arm speed. Individuals with longer arms naturally achieve greater arm speeds because they cover more distance during each reach. Arm length was calculated by summing the distance between the hand and elbow markers and the distance between the shoulder and elbow markers (Fig. 3a). The median value across samples was used as the participant’s arm length.

Delsys data were resampled to a multiple of OptiTrack frequency (240 Hz). EMG signals were resampled to 1440 Hz, and tri-axial accelerometer signals were resampled to 240 Hz.

We calculated EMG amplitude by first subtracting the baseline and applying a bandpass filter with cutoff frequencies of 20–500 Hz, followed by a 60 Hz notch filter to eliminate power line noise. The signal envelope was then computed using a root mean square (RMS) operation applied with a sliding window of 0.05 s and a step size of 1/240 s, resulting in an output signal sampled at 240 Hz.

**Fig. 4:**
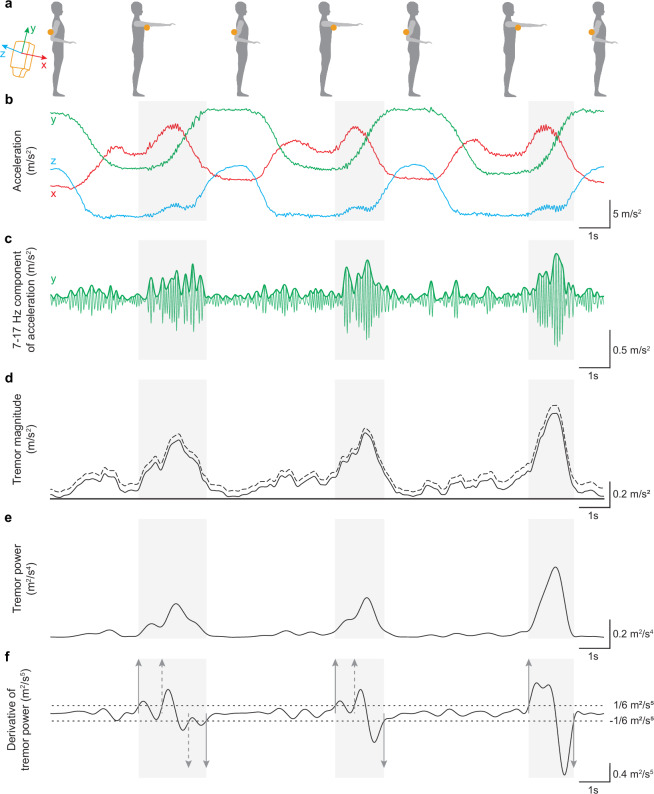
Tremor power calculation and tremor event detection from tri-axial accelerometry. Tremor onsets and offsets were identified using threshold crossings on the time derivative of tremor power. (**a**) Schematic of the reaching task showing the placement of the tri-axial accelerometer (yellow circle) and the local sensor axis convention. (**b**–**f**) Processing pipeline used to generate the tremor annotations provided in the dataset. Shaded gray intervals indicate detected tremor events (identified in f and shown on all panels for reference). (**b**) Representative raw accelerometer signals recorded over the triceps during a trial (axes x, y, z corresponding to the sensor’s local coordinate frame). (**c**) Each axis was band-pass filtered (7–17 Hz, Finite Impulse Response (FIR) filter) and its analytic envelope was computed. One axis is shown as an example (filtered trace and envelope). (**d**) Tremor magnitude was computed as the Euclidean norm of the three axis envelopes (dashed trace) and detrended over the full trial using the adaptive iteratively reweighted penalized least squares algorithm (airPLS)^44^ (solid trace). (**e**) Tremor power was defined as the squared tremor magnitude. (**f**) Thresholds (dashed lines) were applied to the derivative of tremor power to determine tremor onset and offset times (arrows). When necessary, spurious detections (e.g., multiple onsets before a single offset, or multiple offsets) were corrected during manual review. Threshold values were fixed per sensor location based on pilot data and applied consistently across participants.

Physiological tremors were detected from 3-axis accelerometer data collected at the triceps, biceps, and palm (Fig. 4). The sensor’s axis convention is illustrated in Fig. 4a. Accelerometer data was processed in each sensor’s local coordinate space. No spatial transformations to other coordinate frames (e.g., the laboratory coordinate frame) were performed (Fig. 4b). Acceleration signals were band-pass filtered between 7–17 Hz using a finite impulse response filter. The Hilbert transform was applied to each axis, and the Euclidean norm of the resulting envelopes was computed (Fig. 4c).

The tremor magnitude signal was de-trended using the adaptive iteratively reweighted penalized least squares (airPLS) algorithm^44^ to separate postural tremor from movement-induced tremor. It was then smoothed with a 0.5 s moving average filter and tremor power was obtained by squaring the magnitude signal before smoothing (Fig. 4d,e).

Tremor onset and offset times were determined by detecting threshold crossings in the time derivative of tremor power. A fixed threshold value was selected based on pilot data from one sensor location. This reference value was then scaled for the other two sensor locations using the square of the ratio of average tremor amplitudes across all participants. The resulting thresholds for the derivative of tremor power were 1/6, 5/48, and 1/4 m^4^/s^5^ for the triceps, biceps, and palm, respectively. Tremor events were visually inspected and manually corrected for spurious detections (Fig. 4f).

Signal processing parameters, such as filter cutoffs and event-detection thresholds, were set using one of three approaches: commonly used values, pilot data, or group data. Commonly used values included a 10 Hz cutoff for filtering motion capture data and a 20–500 Hz bandpass filter for processing EMG data. Most parameters were determined from pilot data, while two specific parameters were based on group data from all 36 participants. For tremor frequency band estimation, we analyzed the power spectral density (PSD) from accelerometer data, fit a third-order polynomial in log-log space, and subtracted this model fit from the normalized PSD to identify the frequency band and peak frequency at each sensor location. For tremor detection thresholds, we determined the threshold for one sensor location (triceps) using data from all participants, then scaled this threshold for other locations based on the square of the ratio of average tremor amplitudes across all participants.

Ultrasound videos were temporally resampled to a uniform frame rate of 60 frames per second using nearest-neighbor interpolation. The original recorded timestamps for each frame are also provided. These can be used to improve the temporal precision of extracted estimates from ultrasound data.

**Fig. 5:**
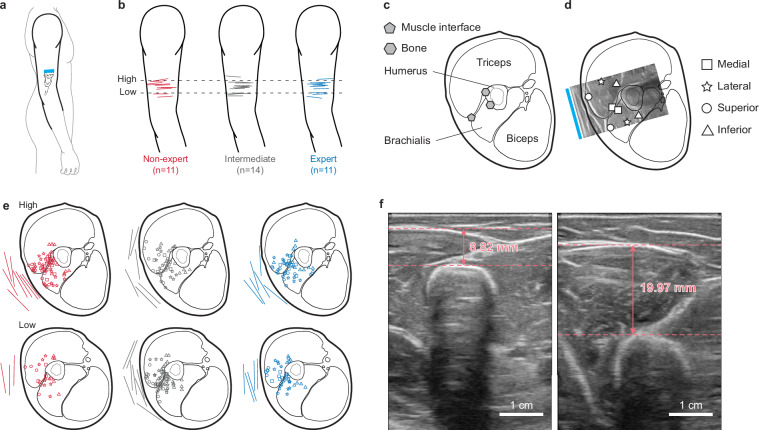
Ultrasound probe placements, locations of tracked points in the upper arm tissues and muscle thickness measurement. (**a**) Schematic of the target ultrasound probe placement (cyan bar) on the upper arm for transverse B-mode imaging of the triceps and brachialis. (**b**) Participant-specific probe placement estimated from photographs by aligning each arm to a common arm template (using shoulder and elbow as reference landmarks) and tracing the probe footprint. (**c,d**) Cross-sectional anatomical sketch illustrating the locations and definitions of the 11 tracked points provided in the dataset. (**c**) Landmark points include one point on the brachialis–triceps interface (gray pentagon) and two points on the humerus (gray hexagons) used to quantify boundary and bone motion within the ultrasound image plane. (**d**) Eight additional intra-muscular points (four per muscle) sample medial (square), lateral (star), superior (circle), and inferior (triangle) regions within the imaged triceps and brachialis. The probe location relative to the cross-sectional diagram (cyan bar) was determined by fitting an ultrasound image taken at the start of the reach cycle to the cross-sectional anatomical reference obtained from The Visible Human Project^45^. (**e**) The probe placement on the circumference of the upper-arm cross section is depicted using lines, and the locations of the tracked points are shown using the symbols defined in d. The red, gray, and blue colors are for participants in the non-expert, intermediate, and expert groups, respectively. (**f**) Representative ultrasound images from two participants illustrating variation in muscle size. Muscle thickness was measured as the distance from the top of the humerus bone to the bottom of the fat layer, with both landmarks visually identified, and represented using horizontal dashed lines. For convenience, in panels b and e, data from participants with left arm probe placements are mirrored.

To verify the consistency of probe placements across participants, we created a sketch of the upper body (Fig. 5a). A picture of the probe placement from each participant was then manually aligned to this sketch using the shoulder and elbow as keypoints, and the position of the probe was traced (Fig. 5b).

To verify the consistency of ultrasound probe placement along the cross-section of the upper arm, we aligned a reference ultrasound image from each participant with a cross-sectional sketch from the Visible Human Project^45^ (Fig. 5c–e). The reference image was captured at the same phase of the reach cycle across all participants (start of the reach cycle). The triceps-brachialis, the fat-muscle, and the humerus-muscle boundaries served as alignment landmarks. Upon alignment, we traced probe locations on the standardized cross-section and spatial distribution of tracked points was mapped (Fig. 5d,e). Of the 11 tracked points, 3 represented consistent landmarks across participants. These included one point at the triceps–brachialis interface that quantifies the muscle boundary motions, and two points on the humerus that can be used to estimate bone motion and rotation with respect to the skin (Fig. 5c). Then, locations of the four points were tracked within each of the triceps and brachialis muscles (Fig. 5d,e). These can be used to estimate muscle deformation and muscle area changes^7^.

The DUSTrack workflow^28^ was used to obtain point trajectories over time in pixel coordinates (Fig. 1e). No transformation was performed to register ultrasound image coordinates to the laboratory (motion capture) reference frame. For each participant, 20–30 frames were manually annotated using a custom graphical user interface designed to promote temporally coherent labeling. Initial labels were augmented using the LK-RSTC algorithm^46^ to generate training data. A DeepLabCut model (ResNet-50 architecture) was trained for 500,000 iterations, and the best snapshot (lowest test error) was used for inference. Model outputs were visually inspected and refined with additional labeled frames if necessary. To reduce frame-to-frame noise from the ResNet outputs, an LK-RSTC based post-processing filter^28^ was used.

In addition to point tracking, the ultrasound video was also used to measure muscle size. A reference image at the start of the reach cycle was used to estimate muscle size as the perpendicular distance from the superior surface of the humerus to the deep boundary of the subcutaneous fat layer. Both anatomical landmarks were identified visually (Fig. 5f).

**Fig. 6:**
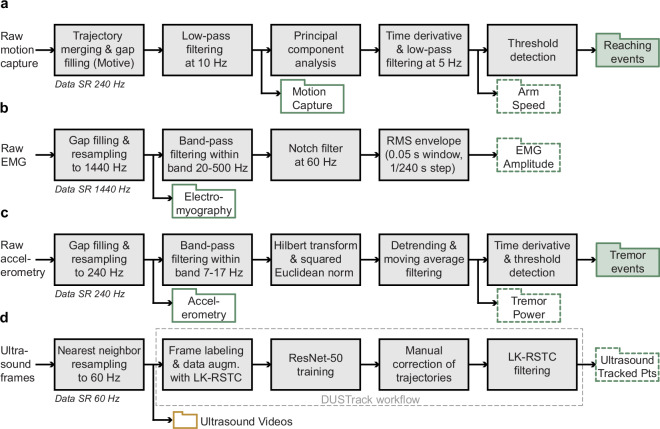
Overview of data-processing pipelines and derived outputs across modalities. Flowchart summarizing the main preprocessing and analysis steps applied to each raw data stream to generate the synchronized signals, derived time series, and event annotations released with the dataset. Solid line filled green folders indicate event groups, solid line folders indicate directly measured data, and dashed line folders indicate derived signals. (**a**) Motion capture processing steps, (**b**) Electromyography processing steps. (**c**) Tri-axial accelerometry processing steps. (**d**) Ultrasound data processing steps. SR stands for sampling rate.

A concise overview of the processing steps applied to each modality is provided in Fig. 6. The flowchart summarizes how raw recordings were cleaned, filtered, resampled, and transformed into the derived signals and event annotations released with the dataset.

### Ethics statement

All participants provided written informed consent to participate and share the data under protocols approved by the MIT Committee on the Use of Humans as Experimental Subjects (COUHES) under the protocol #2201000537. For each participant, we recorded demographic and anthropometric metadata, criteria for expertise categorization, and dominant hand. The board did not provide a consent waiver. All participants provided written informed consent according to the approved protocol. All methods were carried out in accordance with these guidelines and regulations.

## Data Records

The dataset is available on Figshare^40^ and is composed of three primary components: a participant metadata CSV (Comma-Separated Values) file, a collection of synchronized multimodal signals stored in HDF5 (Hierarchical Data Format version 5) files, and a set of corresponding B-mode ultrasound videos (Fig. 6a). In the dataset repository, four additional text files are provided: a list of participant-specific exceptions, a SHA-256 (Secure Hash Algorithm 256-bit) checksum manifest, a description of the HDF5 internal structure, and a README file (Fig. 6a). All HDF5 files are named consistently using anonymized participant IDs (e.g., s14004) and are organized into the structure detailed in the Supplementary Materials. This format supports machine learning workflows and biomechanical analysis pipelines. HDF5 files follow a standardized layout and can be accessed using common programming languages, including Python, MATLAB, and Julia. As standards are still evolving for such multimodal datasets relevant to multiple communities, the dataset is not currently packaged to conform to a specific community standard such as C3D (Coordinate 3D) or NWB (Neurodata Without Borders). The dataset occupies approximately 2 GB of storage.

### Metadata file

The *dataset.csv* file contains demographic and task-related metadata for each participant. Each row corresponds to a single participant and includes fields such as age, gender, dominant limb, anthropometric measures, primary activity, and assigned expertise level (Table 3).

**Table 3:** Metadata fields provided in *dataset.csv*.

Field	Description
*participant_id*	Unique participant identifier, used to link .h5 and .mp4 files
*age*	Participant age in years (yr)
*gender*	Self-identified gender (Male (M), Female (F), or Other (O))
*dominant_side*	Participant dominant limb
*height_cm*	Participant height in centimeters (cm)
*weight_kg*	Participant weight in kilograms (kg)
*arm_length_cm*	Shoulder-to-wrist length in centimeters (cm)
*muscle_thickness_mm*	Muscle thickness at probe site in millimeters (mm)
*primary_activity*	Participant’s main sport or performance background
*expertise_level*	Categorization into expert, intermediate, or non-expert
*probe_side*	Arm used for ultrasound imaging and reaching task
*h5_file*	Path to participant’s HDF5 file
*us_video*	Path to participant’s ultrasound video file

This table includes demographic, anthropometric, and study-configuration information. The *participant_id* serves as the key for linking metadata to the corresponding participant HDF5 recording (.h5) and ultrasound video (.mp4) files. Units and categorical encodings are indicated where applicable.

### Ultrasound videos

**Fig. 7:**
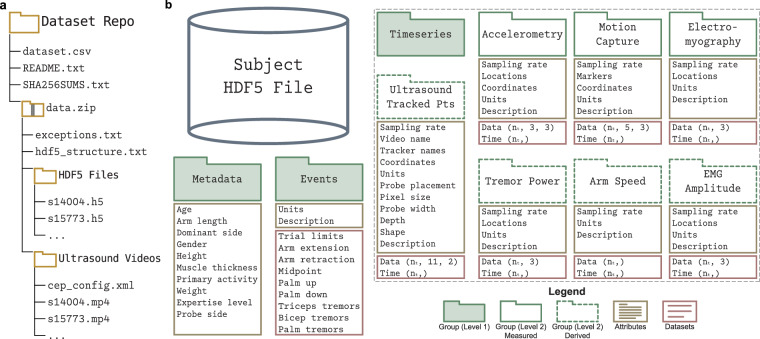
Dataset organization. (**a**) Hierarchical overview of the dataset repository. The top-level directory contains the participant metadata file (*dataset.csv*), a repository *README.txt*, and a SHA-256 checksum manifest (*SHA256SUMS.txt*), along with a compressed archive of the data (*data.zip*). Within *data.zip*, auxiliary text files (*exceptions.txt* and *hdf5_structure.txt*) describe participant-specific exceptions and the HDF5 layout. The archive also contains two subfolders: HDF5 Files (*hdf5_files*), which stores one .h5 file per participant, and Ultrasound Videos (*us_videos*), which stores the corresponding .mp4 ultrasound video for each participant and the Cephasonics config file (*cep_config.xml*). (**b**) Schematic of an HDF5 file and its internal organization. The Metadata (*metadata*) group stores participant and experimental information; the Events (*events*) group lists trial events; and the Timeseries (*timeseries*) group contains all synchronized signals, organized into subgroups: Accelerometry (*acc*), Motion Capture (*mocap*), and Electromyography (*emg*), as direct measures; and Ultrasound Tracked Points (*us_tracked_pts*), Tremor Power (*tremor_power*), Arm Speed (*arm_speed*), and EMG Amplitude (*emg_amplitude*) as derived data. Groups are shown in green, attributes in light brown, and datasets in red. Datasets storing large array tensors indicate their shape in parentheses. Full details of the HDF5 contents are provided in the Supplementary Materials.

Each participant’s ultrasound video is provided as a .mp4 file in the ultrasound videos (*us_videos*) folder (Fig. 7a). These videos are provided at 60 frames per second and capture transverse B-mode views of the upper arm, specifically encompassing the triceps and brachialis muscles throughout the full reaching task. The number of video frames matches the number of samples in the data array of the *timeseries/us_tracked_pts* group within the participant’s HDF5 file. Ultrasound video information and associated metadata are also provided in this group, including frame rate, pixel resolution, probe placement, and physical depth coverage. For reproducibility, the Cephasonics acquisition configuration file (*cep_config.xml*) is also provided in the ultrasound videos folder.

### HDF5 Files

Each .h5 file in the HDF5 folder (*hdf5_files*; Fig. 7a) contains synchronized recordings, derived signals, event annotations, and metadata for a single participant (Fig. 7b). The internal structure adheres to the standard HDF5 format, organized into groups (which function like directories), datasets (which store array data), and attributes (which hold auxiliary information in the form of strings, floats, or integers). A detailed description of the internal structure, including all stored fields, its units, and array shapes, is provided in the Supplementary Materials. Briefly, each file includes a metadata group with demographic and experimental details, which are duplicated from the *dataset.csv* file; an events group that captures the annotated events for each trial; and a timeseries group that stores synchronized sensor data and derived metrics. The timeseries group contains subgroups for direct measures (accelerometry, motion capture, electromyography), and derived signals (ultrasound tracked points, tremor power, arm speed, and EMG amplitude), enabling structured access to the multimodal measurements collected during the reaching task (Fig. 7b).

## Technical Validation

To ensure the reliability, completeness, and physiological plausibility of the dataset, we performed a series of validation and manual verification steps covering data integrity, synchronization accuracy, and signal quality.

### Data completeness and exception handling

All participants were equipped with five optical motion capture markers placed on the hand, forearm, elbow, upper arm, and shoulder; three Delsys wireless sensors placed on the palm, biceps, and triceps; and a transversely oriented ultrasound probe mounted on the upper arm (see Experimental Procedures). Marker placement and sensor configuration were consistent across participants, with few exceptions. Specifically, three participants did not have the forearm marker, and one participant lacked the palm marker due to oversight during data collection. In one participant, the palm Delsys sensor failed mid-experiment, resulting in complete data loss from that sensor. In another participant, a Delsys sensor without EMG recording capability was used on the hand, so only accelerometry data were recorded for that location. Additionally, in four participants, one axis from the accelerometer malfunctioned, recording only the y- and z-axis components of acceleration.

Intermittent sample dropouts occurred in all acquisition modalities. In the motion capture recordings, dropouts primarily arose from brief marker flicker or occlusion. In the Cephasonics ultrasound recordings, they occurred during short periods in which the acquisition frame rate decreased substantially. In the Delsys recordings, dropouts were due to the wireless transmission in the multi-sensor setup. Across all recordings, the mean ± standard deviation percentage of missing samples/frames was 0.310 ± 0.826% for OptiTrack, 0.034 ± 0.021% for Delsys, and 3.592 ± 0.130% for Cephasonics.

For Cephasonics, missing frames were implicitly handled during the resampling process by filling gaps with neighboring frames. For OptiTrack and Delsys, missing samples were marked as NaN and filled using either cubic or linear interpolation via an automated pipeline. In a small number of cases, the automated procedure failed to capture all missing segments. In these cases, they were identified by visual inspection and interpolated manually. All Delsys channels were also screened for amplitude saturation; none was detected.

Ultrasound images were acquired at a variable frame rate (as is typical for this imaging modality) slightly above 60 frames per second on average. During post-processing, videos were resampled to a constant 60 Hz using nearest-neighbor interpolation. This introduced small misalignments between the timestamps of the original frames and the resampled video—an average of 4.6 ± 3.6 ms across recordings.

### Quantification of manual intervention in semi-automated workflows

Several aspects of data processing required manual input. Point tracking in ultrasound videos used the semi-automated DUSTrack workflow^28^. As an illustrative guideline, users of the dataset can expect the average tracking quality to be accurate within a couple of pixels (~100 µm)^28^. This is based on the test error in the deep learning models, as well as perceptual experiments. Further details on the latter and quantitative evaluation of the DUSTrack workflow—including accuracy relative to manual annotations and the accuracy limits imposed by human perception—are provided elsewhere^28^. Each participant’s video was processed by a single annotator, mainly because the point-tracking workflow is highly time intensive^28^.

First, 29 ± 9 frames per participant, typically spanning one reaching cycle, were manually annotated using DUSTrack’s graphical interface. Second, deep learning models were trained, and model predictions were refined by progressively adding training data, typically over 1–3 iterations. Predicted trajectories were then visually inspected across all frames by a second researcher. When trajectory quality was uncertain or insufficient, additional model refinements were performed and the tracking workflow was repeated as needed. Finally, short segments with poor predictions were manually adjusted, often using optical flow tools in DUSTrack^28,46^. On average, manual intervention was applied to 11.94 ± 13.04% of the frames.

Outputs of event-detection algorithms for reach cycle segmentation and tremor detection (see Data Processing) were reviewed through visual inspection. When necessary, synchronized video recordings were used for verification. Manual corrections addressed spurious detections by adding missed events or removing false positives. For extension events, 0.31 ± 0.76% were added on average and 5.50 ± 3.59% were removed. For retraction events, 1.04 ± 1.80% were added on average and 7.22 ± 2.94% were removed. For tremor events, 4.56 ± 4.37% were added on average and 24.79 ± 13.01% were removed.

These manual annotation and verification steps, though time-intensive, were essential to ensure data quality and reliability across all processing stages, thereby strengthening the validity of the dataset for downstream analyses.

### Ultrasound video quality

To provide a quantitative summary of ultrasound video quality across participants, we computed three simple image-based indicators from the exported B-mode videos: the fraction of saturated pixels at the high end of the intensity range (“high saturation”), the fraction of pixels at the low end (“low saturation”), and the equivalent number of looks (ENL), defined as the ratio of the squared mean to the variance, computed within a standardized region-of-interest (ROI) intended to capture relatively homogeneous speckle statistics. For each participant, the ROI was defined as a 40 × 40-pixel window centered on a tracked point within the triceps or brachialis muscle belly.

Across recordings, we report ultrasound video quality indicators as mean ± standard deviation. The percentage of high-intensity saturation was very low (0.0354 ± 0.0372%), indicating that only a small fraction of pixels were clipped at the upper end of the dynamic range. Similarly, low-intensity saturation was minimal (0.0053 ± 0.0077%), suggesting limited floor clipping. ENL values were 48.41 ± 22.75, consistent with relatively homogeneous speckle statistics. Together, these indicators suggest that the released ultrasound videos exhibit minimal intensity clipping and stable texture statistics across participants, supporting downstream use for point tracking, signal processing, and feature extraction.

### Validating task execution

**Fig. 8:**
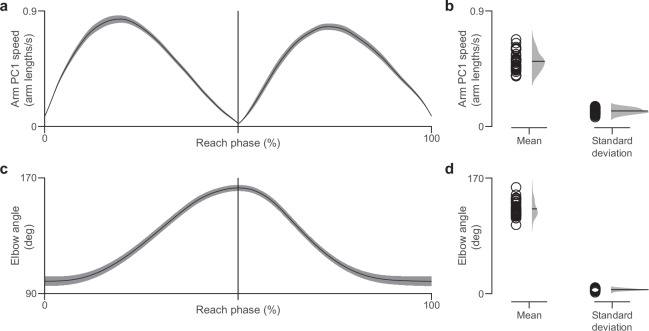
Reach cycle kinematics and variability across participants. (**a**) Average normalized arm PC1 speed (solid line) with standard error of the mean (shaded region), as a function of reach phase across cycles and participants. Values are normalized by arm length. (**b**) Mean and standard deviation of normalized arm PC1 speed across cycle repetitions for each participant. The average value is indicated with a solid horizontal line on distributions. (**c**) Average elbow angle (solid line) as a function of reach phase across cycles and participants with standard error of the mean (shaded region). (**d**) Mean and standard deviation of elbow angle across cycle repetitions for each participant. The mean of each statistic is represented by a solid horizontal line on distributions. We tested for participant-level outliers using Grubbs’ test with Bonferroni correction on the participant means of arm speed (panel b) and elbow angle (panel d). No outliers were detected for either metric: arm speed (*G* = 2.3377 < *G*_crit_ = 2.9906, *p* = 0.6145), elbow angle (*G* = 2.7520 < *G*_crit_ = 2.9906, *p* = 0.1532).

The profile of arm speed (normalized by arm length) was as expected, with two peaks—one at mid-extension and one at mid-retraction—separated by a dip at the phase reversal (Fig. 8a). Elbow angle (derived from shoulder, elbow, and forearm marker positions) increased through extension to a single peak around mid-cycle, then decreased during retraction, remaining within a physiologically plausible range (Fig. 8c).

We assessed whether the task execution was similar within and across participants. The mean and variation across reach cycles were computed for each participant to reveal the inter- and intra-participant variability in our dataset, respectively (Fig. 8b,d). The narrow dispersion of means for both normalized arm speed and elbow angle indicated broadly similar execution across individuals, quantified by an inter-participant coefficient of variation of 0.288 for arm speed and 0.123 for elbow angle. The standard deviation values indicated low intra-subject variation across reach cycles (average intra-participant coefficient of variation of 0.238 and 0.0470 for the arm speed and elbow angle, respectively).

We also used Grubbs’ test with Bonferroni correction to detect outliers in our dataset. This test revealed no outliers among the 36 participants for both the arm speed and the elbow angle (Fig. 8).

### Group-level differences

In addition to validating that participants executed the reaching task in a consistent manner, we assessed whether basic participant characteristics differed across expertise groups, as large demographic or anthropometric imbalances could confound downstream group comparisons. One-way ANOVA tests indicated no statistically significant differences (*p* > 0.05) across the expert, intermediate, and non-expert groups in age, height, weight, or arm length (Table 4). These results suggest that the three expertise groups are broadly comparable in these baseline variables within this cohort. Separately, expertise-related differences in internal tissue dynamics measured from this dataset have been investigated in our companion study^7^, which reports group-level differences in tissue-motion-derived measures.

**Table 4:** Group-level participant characteristics across expertise categories.

Parameter	Expert (n = 11)	Intermediate (n = 14)	Non-expert (n = 11)	*F*_2,33_	*p*
Age (yr)	29.27 ± 8.31	24.86 ± 7.46	24.36 ± 3.84	1.6035	0.2165
Weight (kg)	64.55 ± 11.29	65.36 ± 9.24	66.18 ± 11.62	0.0595	0.9423
Height (cm)	169.82 ± 8.77	173.36 ± 9.53	171.00 ± 7.64	0.4907	0.6166
Arm length (cm)	66.13 ± 3.25	66.49 ± 5.48	65.18 ± 4.22	0.2476	0.7821

Summary statistics (mean ± standard deviation) for age, weight, height, and arm length are reported for the expert, intermediate, and non-expert groups. One-way ANOVA results assess whether each variable differs across groups. No significant group differences were observed for these baseline characteristics (all *p *> 0.05).

### Data integrity and reproducibility

To ensure reproducibility and user confidence, SHA-256 checksums are provided for all data in the dataset, enabling verification of file integrity after download. In addition, all code used to compute derived signals, such as the arm speed from motion capture data and the tremor power from accelerometry, is included to support reproducibility and facilitate methodological extensions. Example scripts are also provided to assist users in loading and navigating the multimodal dataset structure.

### Limitations

While this dataset provides time-synchronized measurements of external kinematics, muscle activation, accelerometry, and internal tissue motions during reaching, several limitations should be considered when using the data.

One limitation to note is the absence of spatial registration across modalities. Although data streams are temporally synchronized, each modality remains in its native coordinate system: OptiTrack marker trajectories in the laboratory reference frame, Delsys accelerometer signals in sensor-specific local axes, and ultrasound data in image pixel coordinates. Additional calibration required for this was not performed since cross-modal spatial analysis was not a consideration when acquiring this dataset. Therefore, it is not possible to express data from one modality in the reference frame of another—for example, representing internal tissue motion captured via ultrasound in the same coordinate frame as external arm kinematics from motion capture.

Limitations inherent to B-mode ultrasound also apply to the ultrasound data in this dataset. The probe provides a 2D view and tissue structures can undergo out-of-plane motion or appear to move due to small probe shifts relative to the skin, which ultimately impacts the accuracy of the tracked point trajectories.

A further limitation concerns the ecological validity of the task design. The data were collected in a controlled laboratory environment using a slow, metronome-paced reaching task. While this approach is more naturalistic than some motor control studies that restrict joint motion (e.g., shoulder fixation) or confine movement to a 2D plane, it still differs from everyday reaching. Natural reaching typically involves variable speeds, external loads, object interactions, visual targets, and environmental constraints—none of which were present in our protocol.

An additional limitation relates to dataset scale for deep learning applications. While the dataset provides frame-level ultrasound tracking across approximately 300,000 frames and 36 participants, this scale may be insufficient for training deep learning based tracking models without pretraining, data augmentation, or supplementary data sources.

Future work could expand the cohort size, introduce greater task variability, and incorporate cross-modal calibration procedures to enable unified spatial analyses.

## Supplementary information

Supplementary Informations

## Data Availability

The dataset is hosted on Figshare^40^ under a CC-BY 4.0 license and is available at 10.6084/m9.figshare.31030252.

## Code availability

A code repository at https://github.com/RogerPallares/reaching-dataset provides Python and MATLAB scripts for computing derived metrics from measured data and tutorials for loading and visualizing the dataset. The repository includes Python (version 3.10) scripts that demonstrate how to compute derived metrics provided in the dataset. Specifically, the scripts show how to compute: (i) arm speed profiles and the onsets and offsets of reaching-cycle events from motion capture data, (ii) tremor power and tremor onsets and offsets from accelerometer data, and (iii) RMS EMG amplitude envelopes from raw EMG signals. The repository also includes two tutorials—one in Python (version 3.10) and one in MATLAB (version R2024b)—that show how to load the HDF5 dataset, navigate its structure, and visualize time series and event data. The tutorials demonstrate how to overlay tracked points on ultrasound video frames and save the annotated video. Additionally, the scripts used to time-synchronize the different data modalities rely on our Python library (pysampled) and they will be made available upon reasonable request.
