# Supplementary material for: A multimodal biomechanics dataset with synchronized kinematics and internal tissue motions during reaching
Source: Sci Data. 2026 Mar 18;13:709. doi: 10.1038/s41597-026-07019-3 (PMC13149674; doi:10.1038/s41597-026-07019-3)
Supplement: Supplementary file 1 — Supplementary Informations [file 41597_2026_7019_MOESM1_ESM.pdf]

Supplementary Materials for

A multimodal biomechanics dataset with synchronized kinematics and internal tissue motions during reaching

Roger Pallarès-López *et al.*

*Corresponding author. Email: praneeth@mit.edu

This PDF file includes:

Supplementary Section: Structured Comparison of Open Upper-Limb Motion Datasets
Supplementary Section: Sensor Placement Images
Supplementary Section: HDF5 File Description

Structured Comparison of Open Upper-Limb Motion Datasets

We conducted a brief scoping review using controlled keyword queries in Google Scholar and PubMed. The analysis fixes anatomy (upper arm) and task (reaching) while varying sensing-modality constraints, with the aim of identifying broad trends in dataset availability. Approximate query hit counts are reported for comparative scoping purposes only (see Supplementary Table 1). We can observe a clear trend in the literature: datasets relying on external measurements are abundant (Q1), datasets that combine ultrasound with reaching are substantially less common (Q2), and datasets that explicitly target internal tissue dynamics during reaching are rare (Q3).

Supplementary Table 1: Structured scoping review of related reaching datasets. *Approximate hit counts returned by controlled keyword queries in Google Scholar and PubMed (January 19, 2026), fixing anatomy (upper arm) and task (reaching) while varying sensing modalities constraints. Counts are reported for comparative scoping purposes and do not represent exhaustive enumeration or validated datasets.*

Query ID	Question	Key added constraint	Approx. hits	
			Google Scholar	PubMed
Q1	*How many reaching datasets relying only on surface/external measurements?*	OMC / IMU / EMG (i.e., no ultrasound)	10600	1352
Q2	*How many datasets claim ultrasound + reaching + at least one other external sensing modality?*	Ultrasound + ≥1 of OMC / IMU / EMG	2610	54
Q3	*How many datasets explicitly target internal tissue dynamics during reaching?*	Ultrasound only, (i.e., tissue deformation / muscle motion)	143	39

From the retrieved results, we manually screened studies for relevance to upper arm reaching and multimodal data acquisition. A small set of 10 representative studies was then selected to construct a comparative table (Supplementary Table 2). This table provides a brief comparison of dataset characteristics and does not offer exhaustive coverage. Existing open upper-limb datasets are dominated by external sensing modalities and application-driven tasks primarily oriented toward prosthetic control, rehabilitation, or human–robot interaction tasks, with reaching well represented but internal tissue dynamics largely absent. In contrast, our dataset adds synchronized ultrasound-based internal tissue motion aligned with kinematics and EMG during reaching, addressing a gap not covered by current open resources.

Supplementary Table 2. Comparison of representative studies reporting open datasets on upper-limb motion. *Studies are summarized by application domain, sensing modalities, movement types, and sample size.*

Dataset	Main application	Sensing modalities	Movement types	Sample size
Jarque-Bou et al.(2019)	Prosthetic control and neurorehabilitation	EMG and strain gauges	Activities of daily living	22 healthy participants
Han et al. (2019)	Prosthetic control	IMU, EMG, and video	Reach-to-grasp, lift, put down, and retract	1 healthy participant
Jeong et al. (2020)	Brain–computer interfaces	EMG, EEG, and EOG	Arm reaching, hand grasping, and wrist twisting	25 healthy participants
Furmanek et al. (2022)	Prosthetic control and neurorehabilitation	OMC and EMG	Reach-to-grasp	10 healthy participants
Hernández et al. (2023)	Manipulability index prediction	EMG and depth sensor	Arm analytic and functional	20 healthy participants
Mastinu et al. (2023)	Robot interaction	Sensorized glove (IMU, strain, and proximity)	Reach-to-grasp	29 healthy participants
Combettes et al. (2024)	Public health and neurological research	OMC	Arm analytic and functional	16 healthy participants
Rima et al. (2025)	Prosthetic control	IMU, M-mode ultrasound, and dynamometer	Arm reaching	5 healthy participants
Thapa et al. (2025)	Brain–computer interfaces	EEG and EOG	Arm reaching and hand grasping	23 healthy participants
Domenico et al. (2025)	Prosthetic control	OMC, EMG, sensorized glove (kinematic and tactile)	Reach and grasp	10 healthy participants

Sensor Placement Images

To support reproducibility of the experimental setup, we provide representative images documenting sensor and marker placement. These images (Supplementary Figure 1) illustrate the locations of the ultrasound probe, optical motion-capture markers, and Delsys EMG/IMU sensors relative to anatomical landmarks used in this study.

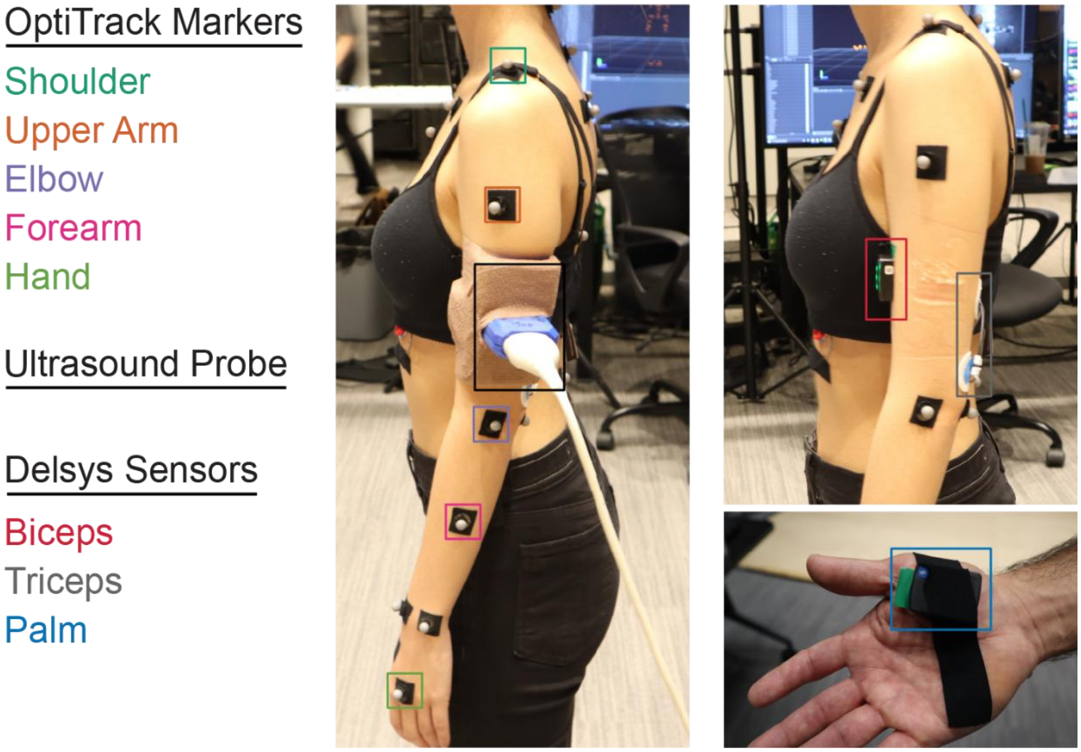

Supplementary Figure 1. Sensor placements and anatomical landmarks. *Representative photographs showing the placement of all sensors used in the reaching task. Left: lateral view of the instrumented arm with five OptiTrack reflective markers placed on the shoulder (acromion, dark green box), upper arm (deltoid insertion, orange box), elbow (lateral epicondyle, purple box), forearm (midpoint between the distal ulna and lateral epicondyle, pink box), and hand (dorsal surface of the 2nd/3rd metacarpal, light green box). The linear ultrasound probe is secured on the upper arm using a custom mount and wrap (black box). Top right: close-up view highlighting the Delsys Trigno sensors on the biceps and triceps (red and gray boxes). Bottom right: close-up of the Delsys sensor on the palm (abductor pollicis brevis, blue box). Colored boxes correspond to the labels shown in the legend on the left. Additional markers/sensors visible in the photographs were used for other concurrent studies and are not included in the released dataset.*

HDF5 File Description

Each *.h5* file contains synchronized recordings, computed signals, event annotations, and metadata for a single participant. The internal structure is organized in the standardized hdf5 format with groups, attributes and datasets, and includes the following information. Words in italic correspond to the actual keys in the files.

- ***metadata***: Group storing subject-level demographic information and experiment-level metadata as attributes. This metadata is duplicated from the corresponding subject row in *dataset.csv* file.
  - Attributes:
    - *age*: Participant age in years (yr).
    - *arm_length_cm*: Shoulder-to-wrist length in centimeters (cm).
    - *dominant_side*: Participant dominant limb (Right or Left).
    - *gender*: Self-identified gender (Male (M), Female (F), or Other (O)).
    - *height_cm*: Participant height in centimeters (cm).
    - *muscle_thickness_mm*: Muscle thickness at probe site in millimeters (mm).
    - *primary_activity*: Participant's main sport or performance background.
    - *weight_kg*: Participant weight in kilograms (kg).
    - *expertise_level*: Categorization into expert, intermediate, or nonexpert.
    - *probe_side*: Arm used for ultrasound imaging and task (Right or Left).
- ***events***: Group containing datasets that define task-related and tremor events as start/end timestamps.
  - Attributes:
    - *units*: Units information for all timestamps. All are in seconds (s).
    - *description*: Description of the event group structure and conventions.
  - Datasets:
    - *extension*: Start and end times of each arm extension phase (shape: number of events x 2) in seconds (s).
    - *midpoint*: Time of the midpoint transition between extension and retraction (shape: number of events x 1) in seconds (s).
    - *palm_down*: Start and end times for the reaching task with the participant's palm facing down (shape: number of events x 2) in seconds (s).
    - *palm_up*: Start and end times for the reaching task with the participant's palm facing up (shape: number of events x 2) in seconds (s).
    - *retraction*: Start and end times of each arm retraction phase (shape: number of events x 2) in seconds (s).
    - *tremor_biceps*: Start and end times of tremor episodes detected at the biceps accelerometer sensor (shape: number of events x 2) in seconds (s).
    - *tremor_palm*: Start and end times of tremor episodes detected at the palm accelerometer sensor (shape: number of events x 2) in seconds (s).
    - *tremor_triceps*: Start and end times of tremor episodes detected at the triceps accelerometer sensor (shape: number of events x 2) in seconds (s).
    - *trial_limits*: Start and end times of the trial (shape: 1 x 2) in seconds (s).
- ***timeseries/acc***: Group storing raw triaxial accelerometer data from three anatomical locations: triceps, biceps, and palm.
  - Attributes:
    - *sr*: Acceleration sampling rate (240 Hz).
    - *locations*: Sensor sites (triceps, biceps, and palm).
    - *coordinates*: Acceleration axis names (x, y, z).
    - *units*: Acceleration units (m/s^2).

        - *description*: Description of the acceleration group structure.
    - Datasets:
        - *data*: Acceleration array (shape: number of samples x 3 locations x 3 axes) in m/s^2.
        - *time*: Time vector in seconds (shape: number of samples).
- ***timeseries/arm_speed***: Group containing the univariate arm speed signal derived from PCA of motion capture data.
    - Attributes:
        - *sr*: Arm speed sampling rate (240 Hz).
        - *units*: Arm speed units (cm/s).
        - *description*: Description of the arm speed group structure.
    - Datasets:
        - *data*: Arm speed array in cm/s (shape: number of samples).
        - *time*: Time vector in seconds (shape: number of samples).
- ***timeseries/emg***: Group storing surface electromyography signals from three muscle sites: triceps, biceps and palm.
    - Attributes:
        - *sr*: EMG sampling rate (1440 Hz).
        - *locations*: Sensor sites (triceps, biceps, and palm).
        - *units*: EMG units (mV).
        - *description*: Description of the EMG group structure.
    - Datasets:
        - *data*: EMG array in mV (shape: number of samples x 3 locations).
        - *time*: Time vector in seconds (shape: number of samples).
- ***timeseries/emg_amplitude***: Group containing the processed amplitude of the EMG signals from three muscle sites: triceps, biceps and palm.
    - Attributes:
        - *sr*: EMG sampling rate (240 Hz).
        - *locations*: Sensor sites (triceps, biceps, and palm).
        - *units*: EMG units (mV).
        - *description*: Description of the EMG group structure.
    - Datasets:
        - *data*: EMG array in mV (shape: number of samples x 3 locations).
        - *time*: Time vector in seconds (shape: number of samples).
- ***timeseries/mocap***: Group storing OptiTrack 3D motion capture trajectories for five upper-limb markers, located at the shoulder, upper arm, elbow, forearm, and hand.
    - Attributes:
        - *sr*: Motion capture sampling rate (240 Hz).
        - *markers*: Marker names (hand, forearm, elbow, upperarm, shoulder).
        - *coordinates*: Marker axis labels (x, y, z).
        - *units*: Marker position units (cm).
        - *description*: Description of the motion capture group structure.
    - Datasets:
        - *data*: Marker position array in cm (shape: number of samples x 5 markers x 3 coordinates).
        - *time*: Time vector in seconds (shape: number of samples).
- ***timeseries/tremor_power***: Group containing processed tremor power signals derived from band-pass filtered accelerometry on the triceps, biceps, and palm.
    - Attributes:
        - *sr*: Tremor power sampling rate (240 Hz).
        - *locations*: Sensor sites (triceps, biceps, and palm).

        - *units*: Tremor power units (m^2/s^4).
        - *description*: Description of the tremor power group structure.
    - Datasets:
        - *data*: Tremor power array in m^2/s^4 (shape: number of samples x 3 locations).
        - *time*: Time vector in seconds (shape: number of samples).
- ***timeseries/us_tracked_pts***: Group containing tracked 2D landmark coordinates over the ultrasound frames.
    - Attributes:
        - *sr*: Trackers sampling rate (60 Hz).
        - *video_name*: Filename of the corresponding .mp4 ultrasound video.
        - *tracker_names*: Name list of tracked points ('br' stands for brachialis, 'tr' stands for triceps).
        - *coordinates*: Tracker coordinate names (x, y).
        - *units*: Tracker position units (mm).
        - *probe_placement*: Side of the body where the probe was placed.
        - *pixel_size_mm*: Pixel size in millimeters (mm).
        - *probe_width_mm*: probe width in millimeters (mm).
        - *depth_mm*: Imaging depth in millimeters (mm).
        - *shape*: Resolution of the ultrasound image (height x width).
        - *description*: Description of the ultrasound trackers group structure.
    - Datasets:
        - *data*: Tracked landmarks position array in mm (shape: number of frames x 11 trackers x 2 coordinates).
        - *time*: Time vector in seconds (shape: number of frames).
        - *time_orig*: Original time vector (non-constant sampling rate) in seconds (shape: number of frames).
